# Simulated fine-needle aspiration diagnosis of follicular thyroid nodules by hyperspectral Raman microscopy and chemometric analysis

**DOI:** 10.1117/1.JBO.27.9.095001

**Published:** 2022-09-07

**Authors:** Marcos A. Soares de Oliveira, Michael Campbell, Alaa M. Afify, Eric C. Huang, James W. Chan

**Affiliations:** a University of California Davis, Department of Pathology and Laboratory Medicine, Sacramento, California, United States; b University of California Davis, Department of Surgery, Sacramento, California, United States; c University of Washington, Department of Laboratory Medicine and Pathology, Seattle, Washington, United States

**Keywords:** hyperspectral Raman imaging, Raman spectroscopy, chemometric analysis, follicular thyroid neoplasm, thyroid cancer

## Abstract

**Significance:**

Follicular thyroid carcinoma carries a substantially poor prognosis due to its unique biological behavior and less favorable outcomes. In particular, fine-needle aspiration (FNA) biopsies, which play a key role in screening thyroid nodules, cannot differentiate benign from malignant follicular neoplasm.

**Aim:**

We report on the use of hyperspectral Raman microscopy in combination with chemometric analysis for identifying and classifying single cells obtained from clinical samples of human follicular thyroid neoplasms.

**Approach:**

We used a method intended to simulate the FNA procedure to obtain single cells from thyroid nodules. A total of 392 hyperspectral Raman images of single cells from follicular thyroid neoplasms were collected.

**Results:**

Malignant cells were identified based on their intrinsic Raman spectral signatures with an overall diagnostic accuracy of up to 83.7%.

**Conclusions:**

Our findings indicate that hyperspectral Raman microscopy can potentially be developed into an ancillary test for analyzing single cells from thyroid FNA biopsies to better stratify “indeterminate” nodules and other cytologically challenging cases.

## 1 Introduction

Thyroid nodules are very common, and ultrasound-guided fine-needle aspiration (FNA) cytology is widely accepted as an initial triage test to determine which patients require excision.^1^^,^^2^ The FNA procedure is performed with a thin hollow needle (25 to 27 gauge) attached to a syringe, and the nodule is aspirated and prepared for cytologic evaluation. Though this approach has allowed clinicians to identify many malignant nodules (particularly papillary carcinoma) that required surgery, diagnosing follicular carcinoma using FNA has been problematic based on cytologic features alone.^3^^–^^5^ This creates a problem for clinicians who must decide if surgery is needed to remove the thyroid nodule.

“Follicular neoplasm” is the term recommended by the Bethesda system for reporting thyroid cytopathology (BSRTC) when screening FNA samples containing moderate to high numbers of microfollicles.^6^ The histologic outcome of these nodules can include hyperplastic/adenomatous nodules, follicular neoplasm, and follicular variant of papillary carcinoma.^7^ Follicular neoplasm can be further subclassified into follicular thyroid adenoma (FTA) and Hürthle cell adenoma (HCA), which are benign, and follicular thyroid carcinoma (FTC) and Hürthle cell carcinoma (HCC), which are malignant. FTC is the second most common thyroid malignancy (papillary carcinoma being the most common) and consists of about 15% of all thyroid cancers.^8^ As capsular or vascular invasion is required on histology for a follicular cancer diagnosis, a cytologic interpretation of follicular neoplasm is, therefore, considered “indeterminate,” leading to clinical management conundrums. As the risk of malignancy for follicular neoplasm ranges from 10% to 40%,^6^ the vast majority of these patients with benign nodules will undergo unnecessary invasive surgery to exclude malignancy.

Thyroid surgery has associated clinical consequences. Many patients will be dependent on lifelong thyroid-replacement therapy, and while uncommon, surgical complications can occur, including iatrogenic hypoparathyroidism and vocal-cord paralysis.^9^ For these reasons, several molecular platforms have been developed and offered commercially to help triage “indeterminate” thyroid FNAs.^10^ However, they have limitations,^11^ thus indicating the need for an alternative method that can provide better diagnostic accuracy (DA) and patient safety.

Raman spectroscopy (RS) is a well-established, nondestructive and noninvasive technique that provides intrinsic chemical information about the sample without requiring exogenous labels or stains.^12^^–^^15^ In addition, it can reach subcellular spatial resolutions if implemented into a confocal microscope. Subtle differences in the chemical and structural compositions of healthy and diseased cells can lead to changes in peak intensities or positions in a Raman spectrum, allowing for identification of malignancy. Previously, we investigated the use of hyperspectral Raman microscopy on human thyroid nodules by using the average Raman spectrum over the entire cell and in association with multivariate statistical analysis, principal component analysis (PCA) and linear discriminant analysis (LDA).^16^^,^^17^ We were able to differentiate benign follicular cells from papillary carcinoma cells with 97% DA.^16^ Also, medullary thyroid carcinoma nodules, whose FNA evaluation is still a challenge, were accurately discriminated from benign and papillary carcinoma with 97% and 99% accuracy, respectively.^17^

In this work, we collected clinical samples of thyroid nodules from patients with initial “follicular neoplasm” interpretation, meaning that their diagnoses were considered indeterminate by FNA cytology evaluation. Only patients who underwent surgery, so final pathological diagnoses could be achieved, were used in this study. In a similar fashion intended to simulate the FNA procedure, we performed hyperspectral Raman microscopy on single cells isolated from the nodule and used chemometric modeling, including support vector machine (SVM) and convolutional neural network (CNN), on the Raman spectral data to develop classification models. The DA of each model was evaluated.

## 2 Materials and Methods

### 2.1 Sample Preparation

Tissue samples of human thyroid nodules were obtained from the University of California Davis Medical Center Biorepository under an Institutional Review Board-approved protocol (#218204-51). All patients were consented prior to study enrollment. The thyroidectomies were processed according to routine surgical pathology with hematoxylin and eosin stain, and the original diagnosis was reconfirmed by a second pathologist to ensure quality assurance of the collected samples. Only nodules with sufficient residual tissue after diagnostic sampling were included in the study.

Single cells were obtained from thyroid nodules using a method intended to simulate the clinical FNA procedure. Similar to the clinical method of *in vivo* FNA, a 27-gauge fine needle attached to a syringe was used to aspirate cells from the *ex vivo* samples. The aspirated cells were released into a phosphate buffered saline (PBS) solution containing 4% paraformaldehyde (PFA) for cell fixation. After a few minutes, the cells were washed by centrifugation and resuspended in PBS solution.

Cells were analyzed by pipetting the cell solution onto a 1-in. round quartz coverslip (0.15- to 0.18-mm thickness) mounted in a cell chamber holder and placed onto the stage of the Raman microscope. Cells remained immersed in the PBS solution for the duration of the spectroscopy measurements. Table 1 summarizes the subtypes of thyroid nodules used in this study and the number of single-cell Raman images that were obtained. Each nodule was obtained from a different patient.

**Table 1:** Thyroid nodules subtypes and number of Raman images from single cells.

Nodule subtype	Number of nodules	Raman image of single cells
FTA	2	61
HCA	3	100
FTC	2	70
HCC	4	161
Total	11	392

### 2.2 Hyperspectral Raman Microscopy

**Fig. 1:**
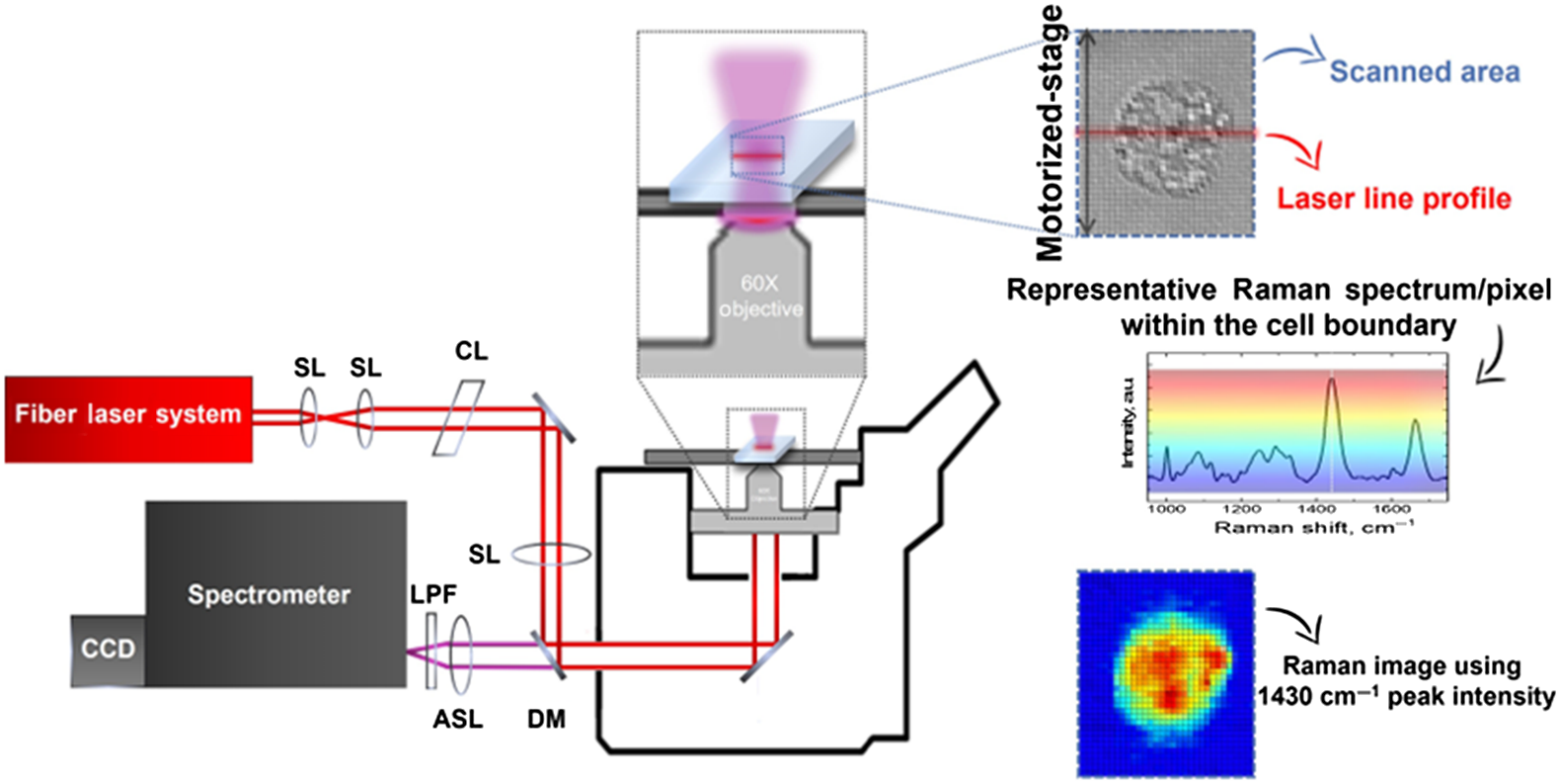
Line-scan hyperspectral Raman microscope setup: scanned area by a line profile laser allowing Raman imaging of single cells. Spherical lens (SL), cylindrical lens (CL), dichroic mirror (DM), longpass filter (LPF), and achromatic spherical lens (ASL).

Hyperspectral Raman images of individual cells were acquired using a previously published method and experimental setup^16^^,^^17^ as shown in Fig. 1. Briefly, a home-built 785 nm Raman microscope with a line-shaped beam profile, from a master oscillator power amplifier fiber laser system (Sacher–laser), at the focal plane of the 60× microscope objective is used. The cell sample is placed on the motorized translational stage of the microscope, which allows the sample to be scanned relative to the laser focus. The Raman signals generated from the line shaped focal region are collected by the microscope objective lens and are imaged by an achromatic lens onto the entrance-slit of the spectrometer and CCD detector. A typical Raman acquisition times per line is 50 s, leading to a full hyperspectral Raman image of a single cell (as shown in Fig. 1) within minutes by scanning the cell with a 1-μm step size in the direction perpendicular to the excitation laser line.

### 2.3 Data Analysis

The multidimensional hyperspectral Raman data of an individual cell is converted into a single Raman spectrum by summing up all Raman spectra belonging to the cell. As such, this spectrum accurately represents the total chemical composition of the entire cell while reducing the data dimensionality. Then, background removal and normalization of the spectrum to the total area under the curve were performed for the purpose of standardizing the dataset.

The combination of PCA and LDA was first used to create a PCA–LDA reference model. The first three PCs were used as inputs for LDA, similar to the previous models used to classify the Raman spectra of different subtypes of thyroid nodules.^16^^,^^17^ PCA is advantageous for feature extraction that maximizes the variance in the dataset, and LDA is suitable for maximizing the separation of the different classes.

Artificial-intelligence-based methods, including SVM and CNN, were then used individually and independently to create classification models by using the full Raman spectra as inputs. For the purposes of optimizing group separation and determining the DA of the Raman spectral signatures, the models are compared. The CNN algorithm model used the limited-memory BFGS (L-BFGS) solver, which is an optimization algorithm of the well-known Broyden–Fletcher–Goldfarb–Shanno (BFGS) quasi-Newton method, and the rectified linear unit (ReLU) as the activation function. The SVM model used the radial basis function (RBF) kernel with a 0.1% numerical tolerance. A random sampling of 95% of the data was performed to train the model, and 5% of the data were used as the test set. For statistical purposes, this process was repeated 100 times, and the prediction of each model was presented in a confusion matrix (CM).

## 3 Results

**Fig. 2:**
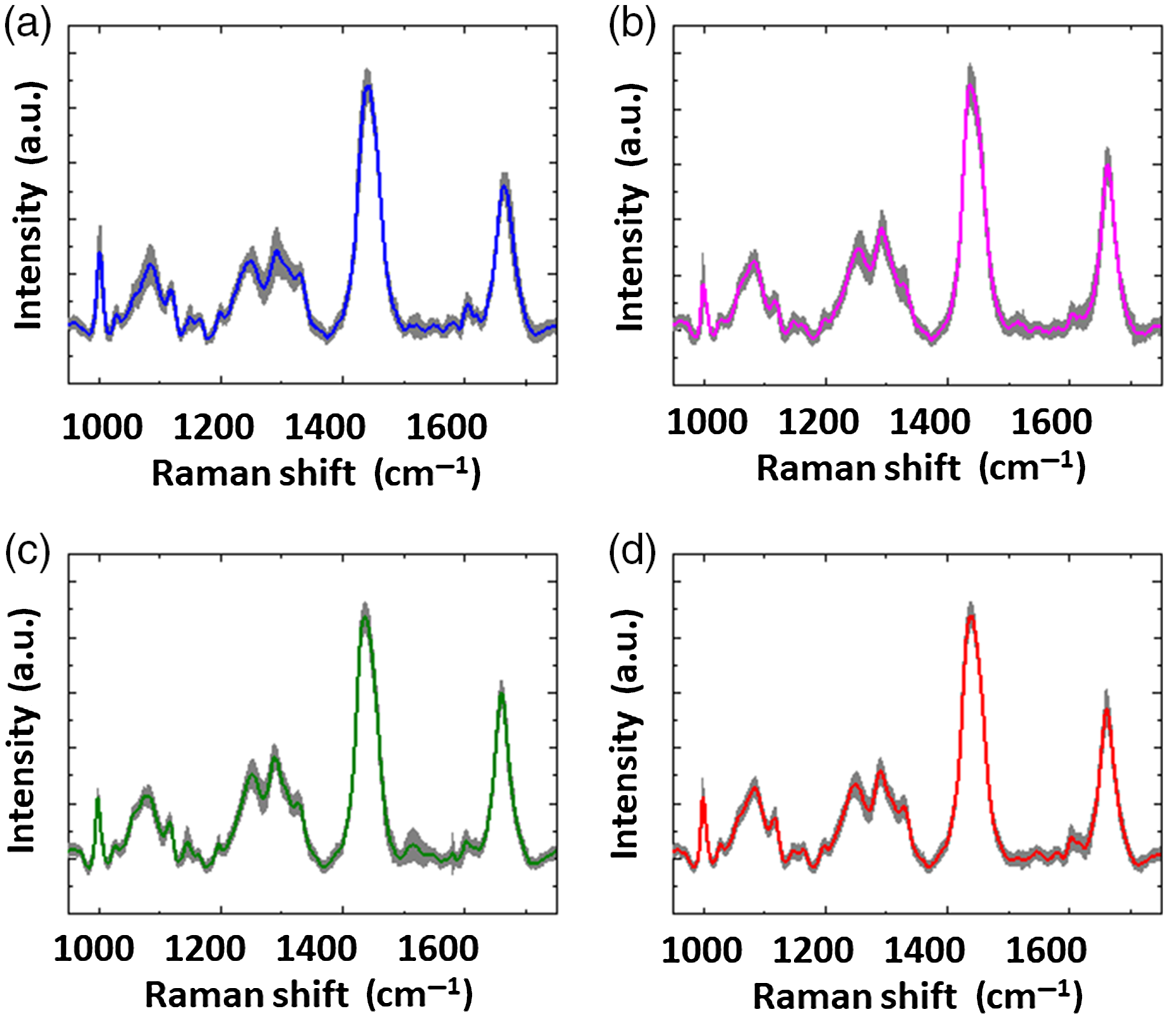
Average Raman spectra of (a) 61 FTA, (b) 100 HCA, (c) 70 FTC, and (d) 161 HCC cells. The gray shadows represent ±1 SD over the average values Raman spectrum of 117 single cells from MTC nodules, with the shaded region representing ±1 SD.

Figure 2 shows the average Raman spectra of 61 FTA (blue line), 100 HCA (green line), 70 FTC (magenta line), and 161 HCC (red line) cells. The light gray shadows represent ±1 standard deviations (SD) over the average values.

**Fig. 3:**
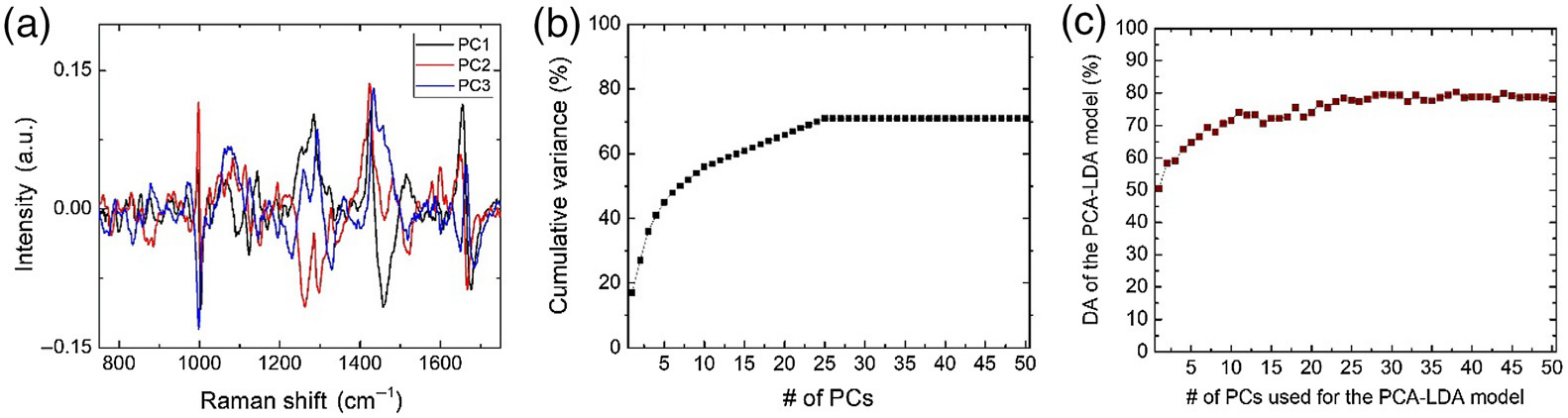
(a) Plot of the first three PCs coefficients. (b) Plot of the cumulative variance as a proportion of the number of PCs. (c) DA of the PCA–LDA model versus the number of PCs used as inputs for the LDA.

Figure 3(a) shows the first three-component coefficients that contribute the most data variance of the Raman spectral features from all four different subtypes used in this study. Figure 3(b) shows the cumulative variance versus the number of PCs used. The first three PCs captured 36% of the total variance in the data. PCA–LDA classification models were then created by using the principal components (PCs) as inputs for LDA, and then a 10-fold leave-N-out cross-validation was used to determine the classification parameters. Each model has an associated-CM; therefore, its associated-DA, which reflects the overall efficiency of the model and is expressed as a proportion of correctly classified subjects among all subjects, can then be calculated. Figure 3(c) shows a plot of the DA of the PCA–LDA model based on the number of PCs used as inputs for the LDA.

The DA of the PCA–LDA model when using the first three PCs was only 59.5%. A higher DA using three PCs for the PCA–LDA model was previously achieved with different thyroid nodule subtypes.^16^^,^^17^ This is an indication of the biochemical similarity of the follicular cells and, consequently, the challenge in differentiating the follicular subtypes in this study. In this paper, we focused on two AI-based methods for the extraction and classification of the Raman spectral features of the follicular cells: SVM and CNN.

**Fig. 4:**
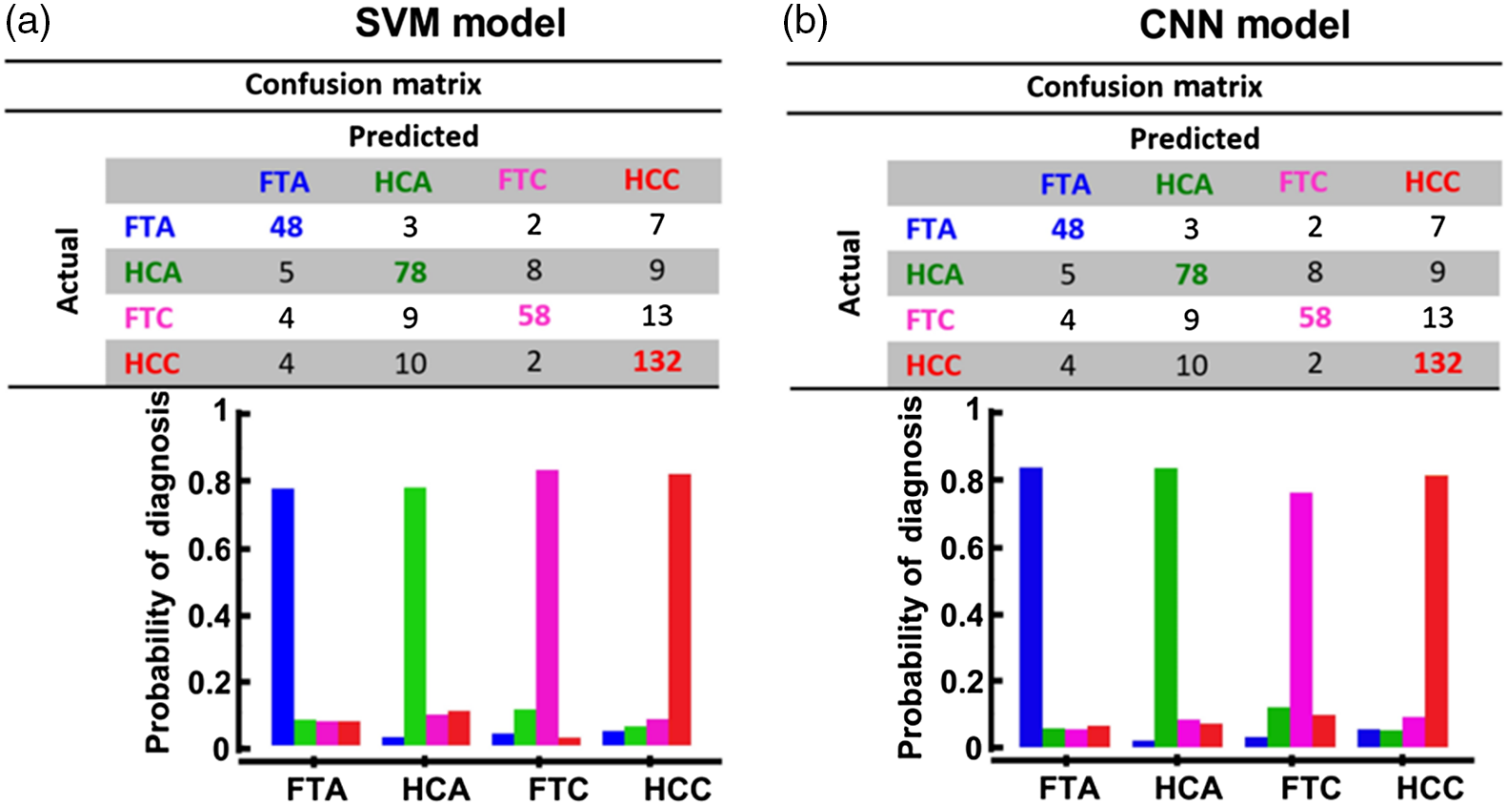
CM and its bar graph distribution of the probability of diagnosis for the (a) SVM and (b) CNN models.

Figure 4 summarizes the CM of each model, in which the probability of accurately diagnosing each follicular thyroid subtype is presented for both AI-based models. The overall DA of the SVM and CNN models, given by the ratio of correctly classified subjects among all subjects, is 80.4% and 80.9%, respectively.

**Fig. 5:**
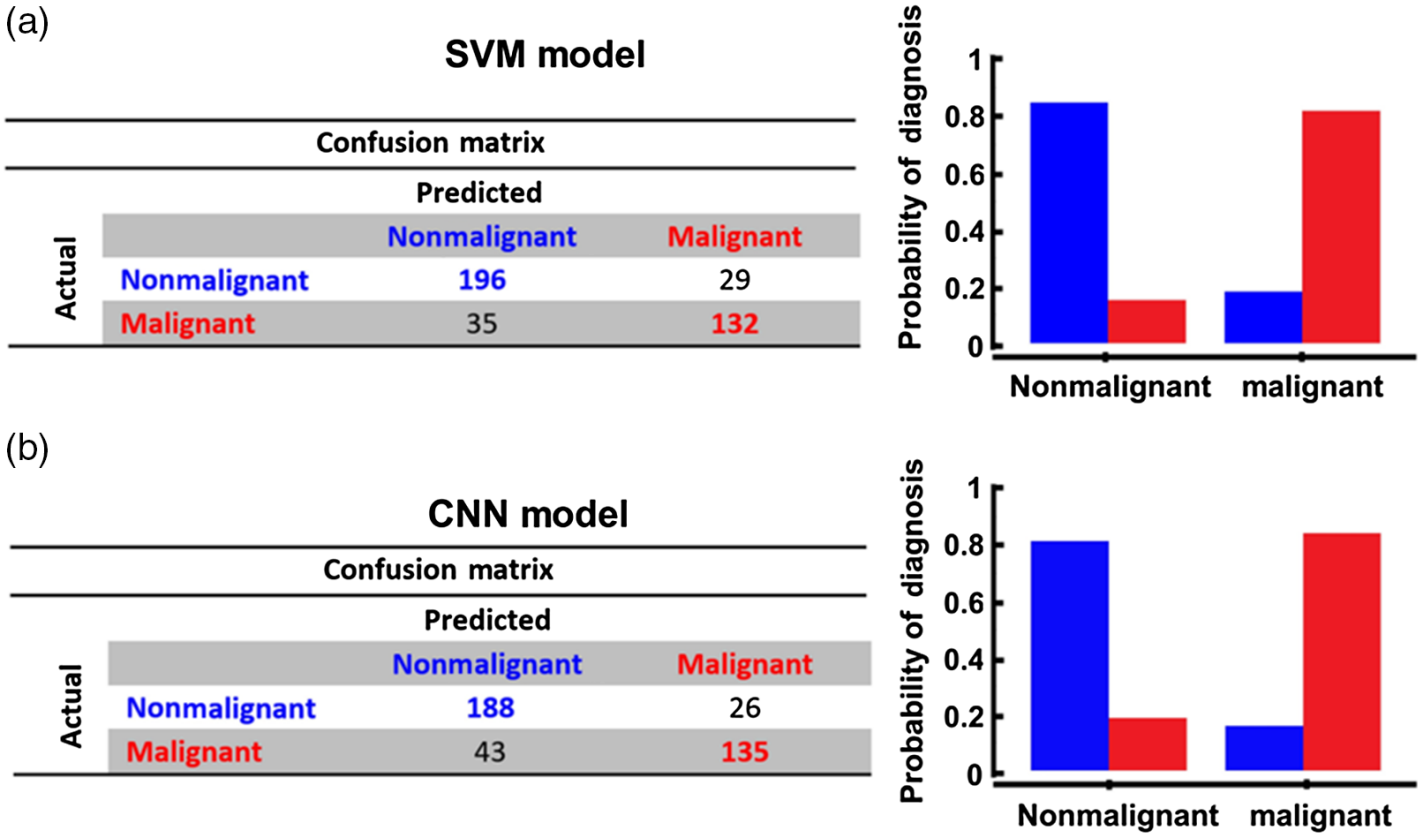
CM and its bar graph distribution of the probability of diagnosis for the (a) SVM and (b) CNN models for discriminating nonmalignant versus malignant follicular nodules subtypes.

Although discriminating each histologic subtype is the most ideal scenario, ultimately, an accurate technique that can distinguish benign from malignant nodules would avoid many unnecessary surgeries and overtreatment. Similar to the previous analyses, Fig. 5 summarizes the probability of accurately diagnosing malignant (including FTC and HCC) and benign (including FTA and HCA) cases.

The overall DA of the SVM and CNN model, given by the ratio of correctly classified subjects among all subjects, is 83.7 and 82.4%, respectively.

## 4 Discussion

FNA cytology has been extremely helpful clinically in screening patients with thyroid nodules, particularly in identifying patients with papillary carcinoma with high sensitivity and specificity. However, follicular neoplasm is diagnostically challenging, as cytologic features alone cannot reliably provide definitive distinction between benign and malignant nodules. Therefore, an accurate minimally invasive methodology is needed to assist nodular thyroid management.

Much progress has been made in the application of ancillary techniques. Recently, some molecular testing platforms (e.g., Afirma GSC & Xpression Atlas, ThyroSeq v.3, and ThyGenX/ThyroMIR) have been developed and are frequently used in conjunction with FNA as an integral part to improve the cytologic evaluation of thyroid nodules.^10^ Molecular tests currently available for thyroid nodules are endorsed by the American Thyroid Association (ATA) for the clinical management of thyroid nodules but still have limited sensitivity and specificity for determining malignancy.

The cytologic interpretation of FTC and HCC is not straightforward. Cytologic evaluation alone cannot distinguish malignant FTC and HCC from their benign counterparts: FTA and HCA. In a recent review, Grani et al.^18^ discussed the challenges of indeterminate thyroid nodules, which also include specifically the challenges in diagnosing FTC and HCC.^5^

With the adoption of new histological classifications, efforts should be made to stimulate additional research in this area, as there is still a need for specific diagnostic and prognostic tools and effective evidence-based surveillance protocols. There has been a push towards developing a novel method that can be widely applied for medical diagnostics;^15^ i.e., the development of a cost-effective minimally invasive single test that can simply and accurately screen and diagnose a wide variety of illnesses and diseases early.

We recently reported using Raman spectral fingerprints to differentiate various thyroid nodules using PCA–LDA models with high sensitivity and specificity.^16^^,^^17^ In fact, the spectral fingerprints of follicular neoplasm subtypes used in this study suggest that there is a higher similarity in their biochemical composition, as more PCs were necessary in this study to achieve higher accuracy, but the spectral feature extraction by the artificial intelligence models used here allowed for a more accurate classification. SVM is a method of discrimination commonly used for pattern recognition in machine learning technology. It is a method in which the data are represented as points in space, so any new data can be mapped into that same space and predicted to belong to a category based on which group they fit better in. CNN is a deep learning method composed of fully connected convolution layers, inspired by neural networks in the brain, in which the features can be automatically extracted and used for classification.

## 5 Conclusion

This study shows that line scan hyperspectral Raman microscopy in combination chemometric, such as AI-based, analysis for cell classification is able to accurately differentiate malignant from benign “follicular neoplasm” nodules prior to surgery. To the best of our knowledge, our work is the first single cell, FNA-simulated, RS study on clinical patient specimens of follicular neoplasms. We believe this approach represent a proof-of-concept study and can be developed into an objective and accurate ancillary tool for analyzing cytology samples to improve diagnostic cytopathology and avoid unwarranted surgeries.

The objective of this study is to provide clinical guidance in managing “follicular neoplasm” and triage patients with a higher likelihood of cancer to surgery. Over the past decade, RS has demonstrated high sensitivity and overall DA when applied to cytologic specimens in various studies. In fact, the application of RS is not unique to thyroid cytopathology, as it has been widely proposed for several clinical applications, including a variety of cancers.^15^ The application of molecular testing for thyroid FNA specimens was accepted in 2015 by the ATA as a follow-up option for thyroid FNAs classified into one of the indeterminate categories.^10^ As we collect a larger cohort, we expect our classification model to improve, which can potentially lead to an alternative ancillary test in assisting the diagnosis and clinical management of thyroid nodules.
